# Ocular Ultrasound in the Diagnosis of Optic Neuropathies: A Review of the Literature

**DOI:** 10.3390/jpm14090949

**Published:** 2024-09-08

**Authors:** Alessia Coppola, Giulia Abbinante, Ilaria De Pascale, Vincenzo Gagliardi, Giulio Salerno, Alfonso Pellegrino, Livio Vitiello

**Affiliations:** Eye Unit, “Luigi Curto” Hospital, Azienda Sanitaria Locale Salerno, 84035 Polla, Italy; ccc.coppolaa@aslsalerno.it (A.C.); ccc.abbinanteg@aslsalerno.it (G.A.); i.depascale@aslsalerno.it (I.D.P.); v.gagliardi@aslsalerno.it (V.G.); gmed.salernog@aslsalerno.it (G.S.); al.pellegrino@aslsalerno.it (A.P.)

**Keywords:** glaucoma, ocular ultrasound, optic nerve, optic neuritis, optic neuropathies

## Abstract

Background: Optic neuropathies represent one of the most frequent causes of vision loss, and they can manifest alone or in conjunction with neurological or systemic symptoms and signs. In recent years, the diagnostic techniques used to detect optic neuropathies have significantly improved, facilitating diagnosis and improving treatment. Among these, ocular ultrasound has assumed a fundamental role, although with conflicting results in the published scientific literature. For this reason, the aim of this review is to analyze the role of ocular ultrasound in the precise and targeted diagnosis of optic neuropathies to better understand the presumed potential of this precious diagnostic tool in the management of these ocular and neurological disorders. Methods: We carried out a search on PubMed and Scopus utilizing terms related to optic neuropathies and ocular ultrasound, including only relevant English full-length research articles, case reports, or case series. Results: Most of the papers published in the scientific literature use only the B-scan ultrasound technique without considering the more precise and objective standardized A-scan technique that allows for performing more accurate diagnostic tests, such as the “30-degree test” and the “optic nerve exercise test”. Conclusions: Future clinical trials and research on optic neuropathies should also consider the use of the standardized A-scan technique in order to compare clinical findings not only with B-scan ultrasonography but also with other noninvasive procedures that could be helpful in reaching the correct diagnosis.

## 1. Introduction

An isolated occurrence of vision loss associated with a more general neurological condition can be caused by disorders of the optic nerve, also known as optic neuropathies. There are many different neurological diseases for which optic neuropathy is frequently the presenting sign, with varied different etiologies [1].

Optic neuropathy is the term used to describe the malfunction and/or degeneration of the optic nerve’s axons, which leads to optic nerve atrophy. Axonal injury and death of retinal ganglion cells are typical features of several optic neuropathies [2] (Table 1).

The two main degenerative causes of damage to the optic nerve are glaucoma and optic neuritis [2].

The site of the damage causing the visual loss is the only thing determined by the diagnosis of the optic neuropathy [3,4]. An optic neuropathy cannot be regarded as a disease unto itself; rather, physicians must first categorize the condition based on the assumed mechanism of the optic neuropathy before trying to use ancillary tests to determine the precise etiology of this pathological condition [5,6]. Understanding the impairments that commonly indicate optic neuropathy and the traits of the various causes aids clinicians in reducing a broad differential diagnosis and carrying out a successful diagnostic evaluation [7].

Ultrasonographic assessment of the optic nerve sheath diameter (ONSD) has been a popular tool for optic nerve examination in recent years [8,9,10]. In addition, ultrasonography is a quick, affordable, safe, non-invasive diagnostic technique that may be quite helpful for bedside evaluations of critically ill patients. However, a well-trained and skilled operator is needed to perform a reliable and well-executed ocular ultrasonography for optic nerve assessment, and several precautions should be taken in the exam execution as well as in the employed probe and technique to obtain trustworthy results [11,12,13,14].

For this reason, the aim of this review is primarily to illustrate the technique of performing ocular ultrasound for the evaluation of the optic nerve and secondarily to analyze the published scientific literature to highlight the potential role of ocular ultrasound in the evaluation of the optic nerve in cases of different optic neuropathies.

## 2. Materials and Methods

We utilized two medical databases, PubMed and Scopus, to perform our search. A preliminary general Google search was also performed in order to have a wider viewpoint. Terms like “ocular ultrasonography”, “point-of-care ultrasound”, “POCUS”, “optic neuropathies”, “glaucoma”, and “optic neuritis” were entered in. The search sentences that were included in the text were either chosen straight from relevant bibliographies or with consideration for the body of current literature. Manual searches of the bibliographies were also carried out in order to find more inclusions. Only English full-length research articles, case reports, or case series pertaining to the ultrasonography assessment of the optic nerve in cases of optic neuropathies were included in this review.

## 3. Ultrasound Technique

Typically, a high-frequency and high-resolution (5–14 MHz) linear array probe is used for B-scan ultrasonography examinations. Generally, the patients are assessed while lying supine, with the head raised approximately 30–40 degrees.

Most clinicians gently apply a standard ultrasonic gel to the closed eyelid and orient the probe at the right angle to observe the optic nerve insertion.

Nevertheless, as the patient’s gaze direction is not visible, this might result in mistakes in the evaluation and measurement of the ONSD and optic nerve. Considering this, skilled ophthalmologists typically perform this diagnostic exam with open lids and with the use of methylcellulose and anesthetic drops, increasing examination accuracy [15,16].

The optic disc is utilized as a reference point for measuring the ONSD from inner edge to inner edge at a distance of 3 mm behind the globe after adjusting the B-scan probe to better view the optic nerve insertion.

Apart from the B-scan examination, “standardized echography” is another well-known ultrasound method in the field of ophthalmology. It is often used with the ultrasonic probe in direct contact with the surface of the eye, combining the A, B, and Doppler methods [15]. This diagnostic method’s uniqueness is directly related to the A-scan instrumentation’s unique design and standardization. In fact, when analyzing the same structure, standardization enables each examiner to acquire the same echograms, resulting in similar and reproducible findings [15]. Moreover, a patient’s age or overall health do not restrict the use of the standardized ultrasonography. However, sedation may be necessary for babies aged six months to three years in order to facilitate a comprehensive and exhaustive examination, while it is performed using only anesthetic drops in adults [15]. Standardized echography can measure the thickness of the optic nerve with an accuracy of +0.3 mm in the retrobulbar space and +0.5 mm in the posterior orbit [15]. Additionally, this ultrasonography method may identify pathological conditions, including meningioma and glioma, as well as neural swelling, increased thickness of the optic nerve sheaths, increased subarachnoid fluid, atrophy, and other disorders [15,17].

Regretfully, B-scan ultrasonography should not be considered an especially objective method of assessing the optic nerve. In fact, when evaluating small anatomical structures, such as the optic nerve, this ultrasound technique, which has been previously evaluated in the literature [18,19], has a number of drawbacks since the B-mode does not have a uniformity of the gain setting [20,21], also known as the “blooming effect” [15,22]. Consequently, this pitfall may be resolved by the standardized A-scan methodology, a blooming effect-free approach that employs an 8 MHz non-focused probe with a customized S-shaped amplification and enables more precise measurements, particularly in the case of the optic nerve assessment [15,16,17,18,19,20,21,22,23].

This method can also avoid the problem of calipers being positioned incorrectly and yield more accurate and exact findings by clearly showing strong reflecting spikes from the interface between arachnoid and subarachnoid fluid [15].

**Figure 1:**
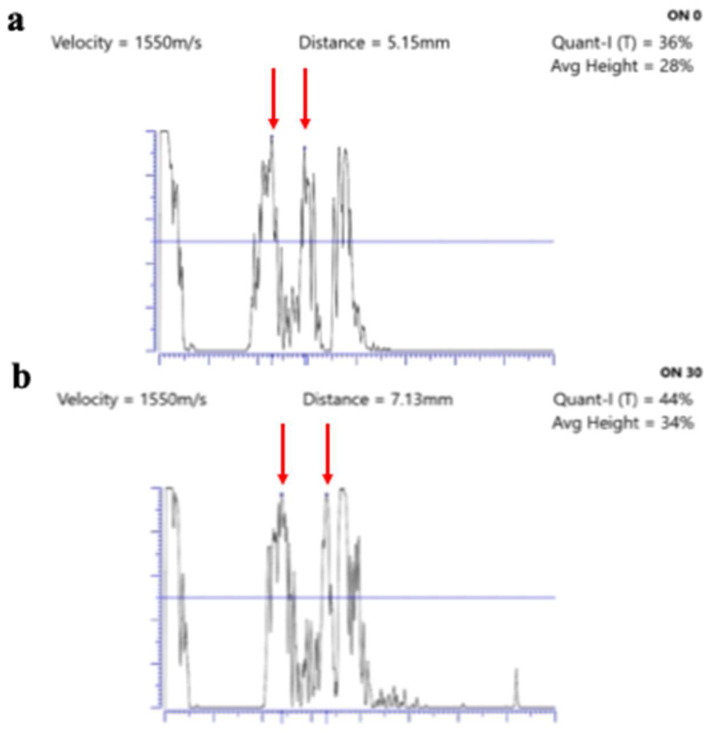
Standardized A-scan ultrasonography of a patient in the primary gaze position (**a**) with an enlargement of the optic nerve (5.15 mm). After the “30-degree test”, (**b**) it is possible to observe the non-reduction in the distance between the hyperreflective peaks (red arrows—7.13 mm), thus demonstrating an increase in the thickness of the optic nerve not related to an increase in intracranial pressure.

In addition, the standardized A-scan examination may be used to perform the “30-degree test” [11]. This test allows for clinicians to differentiate between an increase in ONSD caused by raised intracranial pressure related to increased subarachnoid fluid and an increase in ONSD caused by other disorders, such as optic neuritis or optic nerve meningioma. This maneuver is carried out with the patient looking straight ahead and then to the lateral side in healthy and cooperative patients. If this test demonstrates a decrease in the maximal diameter of at least 5%, it will demonstrate intracranial hypertension caused by increased subarachnoidal fluid, which causes ONSD distension; on the other hand, a solid lesion of the optic nerve or its inflammatory process will not lead to this reduction [11,15] (Figure 1).

Finally, the “optic nerve exercise test” can be used to rule out optic nerve compartment syndrome. In this case, the patient will be asked to look for 15 to 20 s at the very right and left lateral sides in turn throughout this test. This three-minute test is followed by a two-minute eye closure to allow for the subarachnoid fluid—which was driven out of the orbit during the exercise—to return to the baseline position. In healthy people, the orbital subarachnoid fluid frequently returns to its baseline level, unlike what happens in the presence of optic nerve compartment syndrome [14].

## 4. Ocular Ultrasound in Glaucoma

Considering the previously described ultrasound technique [15], it can be used in the same way for the evaluation of the optic nerve in all the optic neuropathies. In particular, for the B-scan, the probe is placed in the temporal portion of the open eye in the primary gaze direction and is tilted until the optic nerve insertion is visualized; on the other hand, when the standardized A-scan technique is performed, the probe is always placed temporally, but this time, the peaks corresponding to the medial rectus muscle are first visualized using it as a reference point. Subsequently, the probe is tilted to identify the peaks corresponding to the optic nerve so that its measurement can be carried out [15]. However, as previously stated, the standardized A-scan technique requires the operator to be skilled in this type of diagnostic evaluation [16,17,18,19,20,21,22,23].

One of the main and most widespread diseases of the optic nerve is certainly glaucoma. Kerlen, in the 1980s, already stated how the optic nerve of glaucomatous patients could be evaluated through excavation of the disc with B-scan ultrasonography, even with media opacities [24]. Conversely, the use of ocular ultrasound in glaucomatous patients to evaluate any other alterations affecting the optic nerve is quite controversial, considering that there are also few published papers on this challenging topic [25,26,27,28].

Abegão Pinto and colleagues [25] were the first to investigate with B-scan ultrasound how ONSD differs between glaucomatous patients and healthy people. They enrolled 46 patients with normal-tension glaucoma (NTG) (patients with optic disc damage and visual field loss), 61 patients with primary open-angle glaucoma (POAG) (patients with untreated intraocular pressure of 21 mmHg or above), and 42 healthy control subjects. They found that ONSD did not significantly differ between healthy, NTG, and POAG patients, with no correlation between visual field damage and ONSD in either of the glaucoma groups. On the other hand, ONSD was found to be correlated with intraocular pressure only in NTG patients. For this reason, the authors hypothesized that the translaminar pressure gradient may play a special role in this particular form of glaucoma [25].

Willekens et al. [26] tried to determine the influence of retrobulbar hemodynamics on ONSD values in glaucomatous patients evaluated by ultrasound color Doppler imaging. In particular, they assessed 88 POAG patients, 58 NTG patients, and 51 healthy controls, and they did not find any significant differences in ONSD between the three study groups, while ONSD was found to be negatively correlated with retrobulbar blood flow velocities in glaucomatous patients but not in healthy controls.

In 2018, Liu et al. [27] studied the optic nerve with ultrasound in 40 NTG patients and 42 POAG patients, comparing them with 46 healthy controls, analyzing the effect that low cerebrospinal fluid pressure in the subarachnoid space of the retrobulbar optic nerve (ONSASA) has on the trans-lamina cribrosa pressure difference in NTG. The results showed that ONSASA was significantly smaller in the NTG group compared to the POAG or control groups, confirming the importance that cerebrospinal fluid pressure has on the trans-lamina cribrosa pressure difference in the NTG etiopathogenesis.

Finally, Omatiga and co-authors [28] evaluated 60 glaucomatous adults and 60 healthy age- and sex-matched individuals to sonographically assess potential changes in the ONSD between the two groups, finding the ONSD of the glaucomatous eyes to be substantially thinner compared to the matching control eyes.

In conclusion, considering the conflicting results and the presence of few clinical studies, further studies are needed to better understand the potential role of the ultrasound ONSD and ocular ultrasonography in the diagnosis and management of glaucoma.

## 5. Ocular Ultrasound in Acute Optic Neuritis

**Figure 2:**
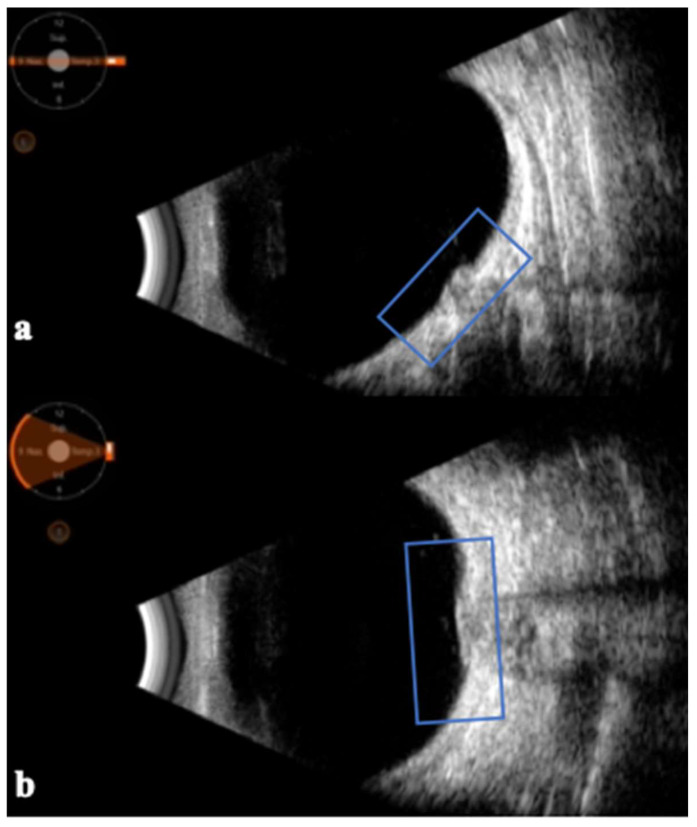
B-scan ultrasonography of the optic nerve insertion in the sagittal (**a**) and transversal (**b**) planes in a patient with papilledema. It is possible to observe the prominence of the optic disc (blue box).

The usefulness of ocular ultrasound in evaluating the optic nerve in cases of inflammatory processes, such as optic neuritis, has been known since the 1980s [15,24,29]. In fact, with B-scan ultrasonography, it is also possible to appreciate the presence of papilledema at the optic nerve insertion, with an elevation of the optic disc (Figure 2).

Several published papers already analyzed the role of ocular ultrasonography in the diagnosis and management of acute optic neuritis.

In the 1990s, a great emphasis was placed on the analysis of the optic nerve with the standardized A-scan method, and Dees et al. [30] performed a pilot study of 27 patients by performing B-scan and standardized A-scan ultrasound on patients presenting with optic neuritis. The 30-degree test was considered to be positive if the swelling decreased by at least 10% on abduction. They classified three groups of patients according to their ultrasound characteristics: normal nerves, swollen nerves with a positive 30-degree test, and swollen nerves with a negative 30-degree test. The results showed a substantial increase in optic nerve diameter in 74% of cases and a positive correlation between nerve swelling and the severity of initial visual loss.

During the same years, Elvin et al. [31] emphasized the importance of Doppler ultrasonography in optic neuritis to determine nerve morphology, nerve swelling, and resistance to flow in the central retinal artery, correlating them with magnetic resonance imaging and visual evoked potentials. The ultrasound examinations were performed on a dynamic scanner with a 7.5 MHz linear transducer within a mean of 27 days of the onset of symptoms. A statistically significant difference was found in the optic nerve diameter and in the resistance to flow in the central retinal artery between the affected and unaffected eyes, confirming the possibility of using Doppler as an indicator of optic nerve inflammation activity.

The possibility of increasing the quality of diagnosis with Doppler ultrasound of the optic nerve was also taken up some years later by Karaali et al. [32] and Karami et al. [33].

The first ones evaluated orbital blood flow velocities with Doppler sonography in 20 patients with acute unilateral optic neuritis, and they found that peak systolic and end diastolic velocities in the ophthalmic artery were significantly increased in the affected orbits.

On the other hand, Karami et al. [33] subverted the conception of Doppler ultrasound for studying the optic nerve because, contrary to the previous studies [31,32], they showed that there were no significant differences in orbital blood flow parameters between the eye with optic neuritis and the healthy contralateral eye of the 23 enrolled patients.

Lochner and colleagues [34,35,36] also utilized B-scan ultrasonography to evaluate the possibility to measure optic nerve diameter and ONSD in patients affected by unilateral optic neuritis, comparing them with healthy controls. They found a statistically significant difference between the two groups in all their clinical research, with a significant thickening of the ONSD in the patients affected by optic neuritis.

In addition, several case reports and case series analyzed the role of ocular ultrasound in detecting optic neuritis in the emergency department [37,38,39].

Wayman and Carmody [37] assessed a 37-year-old diabetic patient complaining of visual loss in the left eye, initially without pain and then with a feeling of pressure on the eye. He underwent a B-scan ultrasonography, which revealed an ONSD dilatation of 6.21 mm and an elevation of the optic disc, both consistent with papilledema or an optic disc swelling. The diagnosis of optic neuritis was then confirmed following an ophthalmological and neurological consultation, also performing a brain magnetic resonance imaging.

Similarly, in two other case series [38,39], the authors were able to make a diagnosis of optic neuritis thanks to the patient’s symptoms and clinical signs and by carrying out an ocular ultrasound with the B-scan method, which revealed a notable thickening of the affected ONSD compared to the healthy contralateral eye.

## 6. Ocular Ultrasound in Optic Neuritis Related to Demyelinating Disorders

Optic neuritis, particularly retrobulbar optic neuritis, can also represent the initial symptom of a central nervous system disease: in particular, demyelinating disorders such as multiple sclerosis [40].

The role of optic nerve ultrasound examination in evaluating retrobulbar optic neuritis was studied in 2005 by Titlić et al. [41] and in 2010 by Stefanović et al. [42].

Titlić et al. [41] prospectively evaluated 20 patients with multiple sclerosis and compared the results with those obtained by magnetic resonance imaging. They found that the diameter of the optic nerve with retrobulbar neuritis showed statistically significant differences from the healthy one and correlated significantly with the number of multiple sclerosis brain lesions.

Stefanović et al. [42], instead, used the B-scan method in 23 patients presenting with retrobulbar optic neuritis and papillitis. Similar to the study performed by Dees et al. [30], they also performed the 30-degree test during the optic nerve assessment. The results showed that the retrobulbar region of the optic nerve was thicker in 94% of the patients with retrobulbar neuritis and in all the patients with papillitis, also finding a correlation between the reduction in visual acuity and the thickening of the retrobulbar region of the optic nerve.

Raeesmohammadi and colleagues [43] sonographically evaluated 60 patients affected by multiple sclerosis who had not recently received an optic neuritis diagnosis, comparing them with 60 healthy sex- and age-matched volunteers. They found that, when optic nerve diameter and ONSD were evaluated by ocular ultrasound, the patients affected by multiple sclerosis who were not currently experiencing an optic neuritis had considerably lower levels than their healthy controls, indicating a gradual subclinical axonal loss.

Similar results were also detected by De Masi et al. [44] and Schroeder et al. [45] who found optic nerve diameter and ONSD evaluated with ocular ultrasound were smaller in patients affected by multiple sclerosis compared to healthy controls, suggesting a chronic depletion of axons in this demyelinating disorder.

Conversely, Elkholy et al. [46] assessed 25 patients with a first attack of an acute demyelinating optic neuritis compared to 25 healthy subjects using ocular ultrasound, showing a significant thickening of ONSD compared to the controls. Furthermore, they also found the ONSD thickness and visual acuity to be significantly inversely correlated.

An increased ultrasound ONSD in the case of patients with optic neuritis related to multiple sclerosis was also found by Saigh et al. [47] and Kwon et al. [48], while Koraysha and colleagues [49] found no significant differences of optic nerve diameter and ONSD between the patients’ group and control group.

In addition, Krogias et al. [50] evaluated 34 optic nerves from 17 patients affected by chronic inflammatory demyelinating polyradiculoneuropathy compared to 30 optic nerves from 15 healthy subjects. Optic nerve diameter and ONSD were sonographically measured, with no significant differences between the two study groups.

Finally, Candeliere Merlicco and co-authors [51] aimed to evaluate the utility of transorbital ultrasonography in the quantification of optic nerve atrophy in multiple sclerosis patients and to determine whether optic nerve atrophy correlates with the disease duration and disability. They enrolled 59 patients diagnosed with relapsing–remitting multiple sclerosis and 36 healthy controls. The ultrasound ONSD was found to be smaller in the patients with multiple sclerosis and to be correlated with the Expanded Disability Status Scale and with the duration of the disease without being interfered by the previous history of optic neuritis.

In conclusion, in cases of demyelinating diseases, the results present in the literature are conflicting, as the thickness of the ONSD measured with ocular ultrasound is variable based on the state of the disease and the presence of acute inflammation of the optic nerve. In fact, in the presence of an acute inflammatory process, the ONSD is increased in thickness, while in the presence of a demyelinating disorder alone, the ONSD is reduced in size compared to healthy controls.

The main findings published in the scientific literature related to optic neuritis are summarized in Table 2.

## 7. Ocular Ultrasound in Other Optic Neuropathies

In 1997, Gerling et al. [52] used ultrasound to study optic nerve diameter in idiopathic optic neuritis and anterior ischemic optic neuropathy. They evaluated 25 patients, 16 with idiopathic optic neuritis and 9 with anterior ischemic optic neuropathy, with a B-scan ultrasound (10 MHz); the probe was placed on the closed upper eyelid while the patient looked temporally. The optic nerve diameter in the patients with optic neuritis with and without disc swelling was significantly larger than that in the contralateral healthy eye. This difference was not confirmed in the patients with anterior ischemic optic neuropathy, and this can be explained by the fact that, in the patients with idiopathic optic neuritis with and without disc swelling, the enlargement of the optic disc is probably caused by an inflammatory edema, which is not present in the case of a vascular optic neuropathy.

Similarly, Dehghani and colleagues [53] evaluated the usefulness of B-scan ultrasonography in distinguishing optic neuritis from non-arteritic anterior ischemic optic neuropathy by measuring the diameter of the optic nerve. They compared 10 consecutive patients with optic neuritis, 10 patients with non-arteritic anterior ischemic optic neuropathy, 10 young controls, and 10 elderly controls. The diameter of the optic nerve was measured using B-scan ultrasonography (13 MHz) by a single radiologist who was not aware of the diagnosis or the side of the lesion. In the optic neuritis patients, the mean diameter of the affected nerve was significantly larger than that of the unaffected nerve and also larger than that of the healthy controls. On the other hand, in the non-arteritic anterior ischemic optic neuropathy patients, there was no significant difference between the mean diameter of the affected nerves and the control group. Moreover, the diameter of the affected nerve was significantly larger in the optic neuritis patients than in the non-arteritic anterior ischemic optic neuropathy patients. For this reason, the authors concluded that B-scan ultrasonography is helpful in the early stages of optic neuropathy to distinguish optic neuritis from non-arteritic anterior ischemic optic neuropathy in those cases for which the diagnosis is still uncertain after clinical evaluation.

Finally, Ji et al. [54] evaluated the ONSD in eyes with dysthyroid optic neuropathy (DON) and its relationship with clinical characteristics and disease severity. A total of 47 healthy eyes, 36 thyroid-associated ophthalmopathy (TAO) eyes without DON, and 33 eyes with DON were studied. All subjects underwent transbulbar B-scan ultrasonography performed by a single investigator using a 10 MHz probe. The authors demonstrated that ONSDs measured at 3 mm and 6 mm of DON eyes were significantly higher than those in non-DON eyes and healthy eyes, showing that ONSD can be potentially used to distinguish DON eyes from non-DON eyes, although the ultrasound ONSD characteristics of chronic DON eyes need to be further examined.

## 8. Discussion

Nowadays, optic neuropathy represents a very dangerous pathological disorder for vision as well as often being the presenting sign of more complex systemic diseases [1,2]. For this reason, a quick and timely diagnosis of this pathological condition could be crucial in order to best manage and treat this clinical entity, thus limiting complications.

This gap may be filled by using ultrasound measurement of ONSD, which has been demonstrated to be a safe, noninvasive bedside diagnostic method that allows for the early start of therapy and the real-time assessment of optic neuropathies. In this therapeutic context, it appears to have unrealized potential even though it is not a revolutionary method [15].

Currently, the biggest pitfall is the substantial variation in ultrasound ONSD reported in the clinical research, as evidenced by conflicting cut-off value results and morphological findings from the published scientific literature pertaining to various optic neuropathies, particularly optic neuritis and glaucoma. Since the B-scan is the diagnostic tool that clinicians use most frequently to assess optic nerves, it is possible that the primary cause of this problem is the lack of uniformity in this ultrasound method. In fact, as previously mentioned in this review, B-scan ultrasonography—which is known to be affected by the “blooming effect” that is connected to the lack of a uniform gain setting—was used in all of the included articles [25,26,27,28,29,30,31,32,33,34,35,36,37,38,39,40,41,42,43,44,45,46,47,48,49,50,51,52,53,54] with the exception of the papers by Dees et al. [30] and Stefanović et al. [42]. Indeed, these papers appear to be the most methodically correct studies because A-scan analysis and the 30-degree test were performed to avoid bias, as previously suggested by Ossoinig [15], even though they are almost 30-year-old and 14-year-old studies, respectively, and they did not analyze a large number of patients.

Considering the “blooming effect”, bias may also have an impact on the calipers’ position when performing ONSD measurements, changing the objectivity and dependability of the results. Furthermore, the B-scan examination was carried out with the patient’s eyelids closed in all of these publications [25,26,27,28,29,30,31,32,33,34,35,36,37,38,39,40,41,42,43,44,45,46,47,48,49,50,51,52,53,54], which might have resulted in mistakes in the evaluation and measurement of ONSD since the patient’s gaze direction was not clearly visible.

For all the above-mentioned B-scan ultrasonography drawbacks, it is recommended to combine the B-scan technique with the standardized A-scan technique [11,12,13,14,15,16,17,18,19,20,21,22,23] to provide data that are as exact, unbiased, consistent, and accurate as feasible. In this way, it is possible to undertake a thorough ultrasound examination with far less bias and no loss of important clinical information, leading to more accurate clinical findings. 

Currently, regardless of the potential usefulness of ocular ultrasound in the evaluation of the optic nerve, its use is still limited in the management of optic neuropathies, considering the conflicting scientific results and that they can also be identified clinically. However, the role of ocular ultrasound can be decisive in distinguishing a thickening of the optic nerve linked to an inflammatory process or to an increase in subarachnoid fluid, thanks to the use of the “30-degree test” previously described [15].

The main limitations of this review are its non-systematic and basically narrative nature and having used only two scientific databases (PubMed and Scopus) for its drafting.

## 9. Conclusions

In conclusion, in order to compare clinical findings not just with B-scan ultrasonography but also with other noninvasive procedures that could be helpful in reaching the correct diagnosis in cases of optic neuropathies, future clinical trials and research carried out on this pathological condition should also utilize the standardized A-scan technique. Furthermore, rather than relying on only ultrasound ONSD, it is crucial to highlight that the assessment of optic neuropathies should be carried out in tandem with accessible data from clinical examinations and other diagnostic techniques, correlating them with each other.

## Figures and Tables

**Table 1:** Differential diagnosis of optic neuropathy.

Glaucoma
Inflammatory - Idiopathic inflammatory optic neuritis (often associated with multiple sclerosis) - Neuromyelitis optica - Systemic inflammatory and autoimmune diseases - Infectious diseases
Vascular - Anterior or posterior - Arteritic or non-arteritic - Post-radiation therapy
Compressive or infiltrative - Neoplastic - Non-neoplastic
Paraneoplastic
Toxic
Nutritional
Hereditary
Traumatic

**Table 2:** Summary of the studies analyzing the role of ocular ultrasound in optic neuritis.

Authors (Year)	Ref.	N. Patients	Ultrasound Technique	Outcomes
Dees et al. (1995)	[30]	27 patients with idiopathic optic neuritis	B-scan and standardized A-scan techniques	Substantial increase in optic nerve diameter in 74% of cases and a positive correlation between nerve swelling and the severity of initial visual loss.
Elvin et al. (1998)	[31]	18 patients with a clinical diagnosis of optic neuritis	Doppler ultrasonography	A statistically significant difference was found in the optic nerve diameter and in the resistance to flow in the central retinal artery between the affected and unaffected eyes, confirming the possibility of using Doppler as an indicator of optic nerve inflammation activity.
Karaali et al. (2003)	[32]	20 patients with acute unilateral optic neuritis	Doppler ultrasonography	Peak systolic and end diastolic velocities in the ophthalmic artery were significantly increased in the affected eyes.
Karami et al. (2012)	[33]	23 patients with acute unilateral optic neuritis	Doppler ultrasonography	No significant differences were found in orbital blood flow parameters between the eye with optic neuritis and the healthy contralateral eye.
Lochner et al. (2017)	[34]	23 patients with unilateral optic neuritis and 19 sex- and age-matched healthy controls	B-scan ultrasonography	Optic nerve sheath diameter and optic nerve diameter are increased in the affected eye.
Lochner et al. (2014)	[35]	21 patients with unilateral optic neuritis and 21 sex- and age-matched healthy controls	B-scan ultrasonography	The median optic nerve sheath diameter and optic nerve diameter were thicker on the affected side compared with the nonaffected side and controls.
Lochner et al. (2017)	[36]	45 patients with newly diagnosed optic neuritis	B-scan ultrasonography	The median optic nerve sheath diameter was thicker on the affected side compared with the nonaffected side.
Wayman and Carmody (2014)	[37]	37-year-old diabetic patient complaining of visual loss in the left eye	B-scan ultrasonography	Optic nerve sheath diameter was found to be 6.21 mm with an elevation of the optic disc. The diagnosis of optic neuritis was then confirmed following an ophthalmological and neurological consultation, also performing a brain magnetic resonance imaging.
Badron and Ong (2019)	[38]	15-year-old girl with optic neuritis	B-scan ultrasonography	Transorbital ultrasound revealed an irregularly enlarged optic nerve sheath with increased optic nerve sheath diameter (5.1 mm) and an elevated optic disc height (0.5 mm). The ultrasound findings correlated well with her magnetic resonance imaging of her orbit.
Yee et al. (2019)	[39]	A 35-year-old man, a 48-year-old man, a 34-year-old female, and a 28-year-old female with optic neuritis	B-scan ultrasonography	Optic nerve sheath diameters of the affected eyes were all found to be more than 5.5 mm.
Titlić et al. (2005)	[41]	20 patients with multiple sclerosis and retrobulbar neuritis	B-scan ultrasonography	The diameter of the optic nerve with retrobulbar neuritis showed statistically significant differences from the healthy one and correlated significantly with the number of multiple sclerosis brain lesions.
Stefanović et al. (2010)	[42]	23 patients presenting with retrobulbar optic neuritis and papillitis	B-scan and standardized A-scan techniques	The retrobulbar region of the optic nerve was found to be thicker in 94% of the patients with retrobulbar neuritis and in all the patients with papillitis, also finding a correlation between the reduction in visual acuity and the thickening of the retrobulbar region of the optic nerve.
Raeesmohammadi et al. (2020)	[43]	60 patients affected by multiple sclerosis who had not recently received an optic neuritis diagnosis, and 60 healthy sex- and age-matched volunteers.	B-scan ultrasonography	Patients affected by multiple sclerosis who were not currently experiencing an optic neuritis had considerably lower levels of optic nerve sheath diameter and optic nerve diameter than their healthy controls, indicating a gradual subclinical axonal loss.
De Masi et al. (2016)	[44]	60 unselected relapse-free multiple sclerosis patients and 35 matched healthy controls	B-scan ultrasonography	Optic nerve diameter and optic nerve sheath diameter evaluated with ocular ultrasound were found to be smaller in patients affected by multiple sclerosis compared to healthy controls, suggesting a chronic depletion of axons.
Schroeder et al. (2020)	[45]	78 patients with a history of demyelinating disorders	B-scan ultrasonography	Optic nerve diameter and optic nerve sheath diameter evaluated with ocular ultrasound were found to be smaller in patients affected by multiple sclerosis compared to healthy controls, suggesting a chronic depletion of axons.
Elkholy et al. (2020)	[46]	25 patients with a first attack of an acute demyelinating optic neuritis compared to 25 healthy subjects	B-scan ultrasonography	A significant thickening of optic nerve sheath diameter was found compared to controls. Furthermore, optic nerve sheath diameter thickness and visual acuity were found to be significantly inversely correlated.
Saigh et al. (2019)	[47]	59-year-old female patient with progressive unilateral visual loss while she was receiving outpatient treatment for relapsing–remitting multiple sclerosis	B-scan ultrasonography	Transorbital ultrasound revealed a disparity between the optic nerve sheath diameters of the affected and nonaffected eyes and striking optic nerve edema in the affected eye, thus diagnosing an acute optic neuritis.
Kwon et al. (2019)	[48]	17 patients with first-attack unilateral acute optic neuritis	B-scan ultrasonography	Ocular ultrasonography revealed thickening of the optic nerve diameter and optic nerve sheath diameter on the affected side compared with the unaffected side.
Koraysha et al. (2019)	[49]	23 patients affected by relapsing–remitting multiple sclerosis with a history of optic neuritis, 26 patients affected by relapsing–remitting multiple sclerosis without a history of optic neuritis, and 50 age- and sex-matched healthy controls	B-scan ultrasonography	No significant differences of optic nerve diameter and optic nerve sheath diameter were found between the patients’ group and control group.
Krogias et al. (2016)	[50]	17 patients affected by chronic inflammatory demyelinating polyradiculoneuropathy compared to 15 healthy subjects	B-scan ultrasonography	Optic nerve diameter and optic nerve sheath diameter showed no significant differences between the two study groups.
Candeliere Merlicco et al. (2018)	[51]	59 patients diagnosed with relapsing–remitting multiple sclerosis and 36 healthy controls	B-scan ultrasonography	The ultrasound optic nerve sheath diameter was found to be smaller in patients with multiple sclerosis and to be correlated with the Expanded Disability Status Scale and with the duration of the disease without being interfered by the previous history of optic neuritis.

## Data Availability

No new data were created or analyzed in this study. Data sharing is not applicable to this article.
